# 

**Published:** 1922-02

**Authors:** 


### ANNOUNCEMENTS

SOCIETY MEETINGS

Michigan State meeting, in Detroit, April 4 to 6.

Kentucky State meeting, in Louisville, April 10 to 12.

West Virginia, at Fairmount, April 10 to 12.

Illinois, in Springfield, May 9 to 11.

Indiana, in Indianapolis, May 15 to 17.

Wisconsin State Board of Examiners will hold examinations for dentists and hygienists, June 19 to 24.

---

First District Dental Society at Detroit, Michigan, at a recent session, passed the following resolution discrediting as unethical the practice of members of the profession for giving or accepting money in payment for services as essayists or clinicians, as follows:

*Resolved,* That the First District Dental Society, of Michigan, shall hereafter consider the payment of fees for the imparting of scientific knowledge as contrary to our professional ethics, and not in keeping with the high ideals we endeavor to maintain.

---

The Texas State Dental Society will hold its Forty-second Annual Convention March 13 and 14, 1922, at Houston, Texas.

The special features of the program will be lectures and clinic work by Dr. Boyd S. Gardner, of Rochester, Minn., and Dr. Carl Hoffer, of Nashville, Tenn., with symposiums and unit clinics by members of the society.

During the four days following, a post-graduate class course will be conducted by Drs. Willis A. Coston, Topeka; Russell W. Tench, New York City; Arthur E. Smith, Chicago; T. W. Maves, Minneapolis, and Julian Smith, Dallas.—J. G. Fife, *Secy.*, 1813 Main St., Dallas, Texas.

The next Annual Meeting of THE KENTUCKY STATE DENTAL ASSOCIATION will be held in Louisville, Ky., April 10, 11, 12, 1922, Seelbach Hotel as headquarters. A clinical program of unusual interest is being arranged. Address all correspondence to W. M. Randall, *Secy.*, Louisville, Ky.

---

The next TENNESSEE STATE DENTAL SOCIETY meeting will be held in Memphis, Tenn., on May 8, 9, 10, 11, 1922. —Camilla N. Mays, *Secy.*

---

THE DENTAL SOCIETY OF THE STATE OF NEW YORK will hold the Fifty-fourth Annual Meeting at Convention Hall, Rochester, N. Y., May 11, 12, 13, 1922. All literary exercises, clinics and exhibits will be stationed at Convention Hall.

Officers' headquarters will be at Hotel Seneca.

The society extends a cordial welcome to all ethical dentists of New York and sister states. The preliminary program will be issued about April 10th, and the official program May 1st. Dentists not members of the state society may obtain program by addressing the secretary. Make hotel reservations early. Hotels: Seneca, Powers, Rochester, Hayward, etc.

A cordial invitation is extended to exhibitors. Address Dr. John J. Scott, 905 Commerce Building, Rochester, N. Y. For further information, address A. P. Burkhart, *Secy.*, 89 Genesee St., Auburn, N. Y.

### Industrial Companies which have completed their medical service by the addition of a dental department

THERE have been many requests for our printed list of companies using Dental Service. The book "Modern Dentistry for the Laity" will soon appear with a corrected and up-to-date list of all such companies.

The following is the list as it appears at the time of going to press. Get your order in early for a copy of the new edition of "Modern Dentistry for the Laity."

**Adamsville, Alabama—**
Docena Iron Works.
Tennessee Coal & Iron Co., Wenonah Mines.
Tennessee Coal & Iron Co., Ishkooda Mines.

**Acipo, Alabama—**
American Cast Iron Pipe.

**Alabama City, Alabama—**
Dwight Mfg. Co.

**Birmingham, Alabama—**
American Cast Iron Pipe Co.
Tennessee Coal & Iron Co.

**Bessemer, Alabama—**
Tennessee Coal & Iron Co., Muscoda Works.

**Chickasaw, Alabama—**
Chickasaw Shipbuilding Co.

**Ensley, Alabama—**
Tennessee Coal & Iron Co., Bayview Mines.
Westfield Clinic.

**Edgewater, Alabama—**
Edgewater Works.

**Fairfield, Alabama—**
Tennessee Coal & Iron Co.

**Johns, Alabama—**
Tennessee Coal & Iron Co.

**Davenport, California—**
Santa Cruz Portland Cement Co.

**Fresno, California—**
California Associated Raisin Growers.

**San Francisco, California—**
The Emporium.

**Denver, Colorado—**
The Colorado Fuel & Iron Co.
Daniels & Fisher Store Co.
Gates Rubber Co.

**Pueblo, Colorado—**
The Colorado Fuel & Iron Co.

**Trinidad, Colorado—**
The Colorado Fuel & Iron Co.

**New Haven, Connecticut—**
Winchester Repeating Arms Co.

**Milford, Delaware—**
The L. D. Caulk Co.

**Wilmington, Delaware—**
Dupont Powder Co.
Joseph Bancroft & Sons Co.
Electric Hose Co.

**New Haven, Connecticut—**
Kolynos Co.
L. Candee Co.

**Stamford, Connecticut—**
Yale & Towne Mfg. Co.

**South Manchester, Connecticut—**
Cheney Bros. Co.

**Atlanta, Georgia—**
Fulton Bag & Cotton Mills.

**Chicago, Illinois—**
Morris & Co.
Montgomery & Ward Co.
Armour & Co.
The Crane Co.
The Diamond Match Co.
Hart, Schaffner & Marx.
Kabo Corset Co.
Sears, Roebuck & Co.
The International Harvester Co.

**Peoria, Illinois—**
The Avery Co.

**Indiana Harbor, Indiana—**
The Inland Steel Co.

**New Orleans, Louisiana—**
D. H. Holmes Co.
Maison Blanche, Inc.

**South Brewer, Maine—**
The Eastern Mfg. Co.

**Boston, Massachusetts—**
The Hood Rubber Co.
Filene Co-operative Association.

**Watertown, Massachusetts—**
Hood Rubber Co. (Factory).

**Framingham, Massachusetts—**
The Dennison Mfg. Co.

**Chicopee Falls, Massachusetts—**
Fisk Rubber Co.
A. G. Spalding Co.

**Pittsfield, Massachusetts—**
General El. Co.

**Springfield, Massachusetts—**
Am. Bosch Magneto Co., Brightwood Station.
National Equipment Co.

**Holyoke, Massachusetts—**
Farr Alpaca Co.

**Lynn, Massachusetts—**
General Electric Co.

**Worcester, Massachusetts—**
The Norton Co.

**Wekter, Massachusetts—**
S. Slater & Sons Co.

**Wellesly Hills, Massachusetts—**
Babson Statistical Organization.

**Detroit, Michigan—**
The Ford Motor Car Co.
Solvay Process Co.

**Kalamazoo, Michigan—**
Kalamazoo Stove Co.

**Minneapolis, Minnesota—**
The Washburn-Crosby Co.
The Northwestern Knitting Co.
National Lamp Co.
Munsingwear Corporation.

**Kansas City, Missouri—**
Jones Store Co.
Montgomery & Ward.
National Lamp Works.

**St. Louis, Missouri —**
National Lamp Works.
Union Electric Light & Power Co.
Monsanto Chemical Works.
Stix, Baer & Fuller Dry Goods Co.
Century Electric Co.
B. Nugent & Bros. Dry Goods Co.

**Concord, New Hampshire—**
Union District Dental Clinic.

**Manchester, New Hampshire—**
The Amoskeag Co.

**Nashua, New Hampshire—**
Nashua, City Dental Clinic.
Maginnis Cotton Mills.

**Harrison, New Jersey—**
Harrison Lamp Works.
The General Electric Co.

**Hurley, New Mexico—**
Chino Copper Co.

**Brooklyn, New York—**
Schrader & Sons.

**Buffalo, New York—**
Larkin Co.
American Radiator Co.
Buffalo Miniature Lamp Works.
Pierce-Arrow Motor Car Co.

**Johnson City, New York—**
Endicott-Johnson Co.
E.-J. Workers Medical & Relief.

**McGregor, New York—**
Metropolitan Life Insurance Co.

**New York City, New York—**
International Garment Workers' Union.
Lord & Taylor.
Macy Mutual Aid Assn.
James McCreery & Co.
Metropolitan Life Insurance Co.
New York Telephone Co.
John Wanamaker Store.
Colgate & Co.
Sanatorium.

**Niagara Falls, New York—**
Oldsbury Electro Chem. Co.

**Rochester, New York—**
The Bousch & Lomb Co.

**Schenectady, New York—**
General Electric Co.

**Akron, Ohio—**
The Firestone Tire & Rubber Co.
The B. F. Goodrich Co.

**Cincinnati, Ohio—**
The Cincinnati Milling Machine Co.
The R. K. LeBlond Machine Tool Co.
The Worthington Pump Co.
The Lunkenheimer Co.

**Cleveland, Ohio—**
The Bailey Co.
The Joseph & Feiss Co.
The Kaynes Co.
National Lamp Works of General El. Co. (Parent Co. of all Natl. Lamp Works.)
H. & E. Blouse Co.

**Columbus, Ohio—**
Jeffries Mfg. Co.

**Dayton, Ohio—**
Dayton Electric Laboratories Co.
The Dayton Metal Products Co.
The National Cash Register Co.
Domestic Engineering Co.

**Hamilton, Ohio—**
The Hooven-Owens-Rentschler Co.

**Newcomerstown, Ohio—**
J. B. Clow Co.

**Warren, Ohio—**
National Lamp Works of General El. Co.

**Youngstown, Ohio—**
National Lamp Works of General El. Co.

**Erie, Pennsylvania—**
Erie Forge Co.

**Philadelphia, Pennsylvania—**
Powers, Weightman, Rosengarten Co.
John Wanamaker.
The S. S. White Dental Mfg. Co.
Lit Brothers.
J. G. Brill Co.
John B. Stetson Co.
The American Pulley Co.

**Pittsburgh, Pennsylvania—**
The Armstrong Cork Co.
The H. J. Heinz Co.
Lee S. Smith & Son Co.
Kaufmann's, The Big Store.
Kauffmann's, "The Narrow Fabric Co."

**Reading, Pennsylvania—**
Berkshire Knitting Mills.
Berkshire Knitting Machine Co.

**Bristol, Rhode Island—**
National India Rubber Co.

**Central Falls, Rhode Island—**
Rhode Glass Division (Natl. Lamp Works, General Electric Co.).

**Esmond, Rhode Island—**
Esmond Mills.

**Bosevaine, Virginia—**
Pocahontas Fuel Co.

**Itman, West Virginia—**
Pocahontas Fuel Co.

**Jenkinjones, West Virginia—**
Pocahontas Fuel Co.

**Maybeury, West Virginia—**
Pocahontas Fuel Co.

**War, West Virginia—**
Williams Pocohontas Coal Co.

**Appleton, Wisconsin—**
Kimberly-Clark Co.

**Kohler, Wisconsin—**
Kohler Mfg. Co.

**Milwaukee, Wisconsin—**
Phoenix Knitting Mills.
Cutler-Hammer Mfg. Co.
Chain Belt Co.
Wisconsin Bridge Works.

**Neenah, Wisconsin—**
Kimberly-Clark Co.

**Niagara, Wisconsin—**
Kimberly-Clark Co.

**Toronto, Canada—**
T. Eaton Co., Ltd.

